# Canal-Net for automatic and robust 3D segmentation of mandibular canals in CBCT images using a continuity-aware contextual network

**DOI:** 10.1038/s41598-022-17341-6

**Published:** 2022-08-05

**Authors:** Bo-Soung Jeoun, Su Yang, Sang-Jeong Lee, Tae-Il Kim, Jun-Min Kim, Jo-Eun Kim, Kyung-Hoe Huh, Sam-Sun Lee, Min-Suk Heo, Won-Jin Yi

**Affiliations:** 1 Interdisciplinary Program in Bioengineering, Graduate School of Engineering, Seoul National University, Seoul, Korea; 2 Department of Applied Bioengineering, Graduate School of Convergence Science and Technology, Seoul National University, Seoul, Korea; 3 Vision AI Business Team, LG CNS, Seoul, Korea; 4 Department of Periodontology, School of Dentistry and Dental Research Institute, Seoul National University, Seoul, Korea; 5 Department of Electronics and Information Engineering, Hansung University, Seoul, Korea; 6 Department of Oral and Maxillofacial Radiology and Dental Research Institute, School of Dentistry, Seoul National University, Seoul, Korea

**Keywords:** Oral anatomy, Machine learning

## Abstract

The purpose of this study was to propose a continuity-aware contextual network (Canal-Net) for the automatic and robust 3D segmentation of the mandibular canal (MC) with high consistent accuracy throughout the entire MC volume in cone-beam CT (CBCT) images. The Canal-Net was designed based on a 3D U-Net with bidirectional convolutional long short-term memory (ConvLSTM) under a multi-task learning framework. Specifically, the Canal-Net learned the 3D anatomical context information of the MC by incorporating spatio-temporal features from ConvLSTM, and also the structural continuity of the overall MC volume under a multi-task learning framework using multi-planar projection losses complementally. The Canal-Net showed higher segmentation accuracies in 2D and 3D performance metrics (p < 0.05), and especially, a significant improvement in Dice similarity coefficient scores and mean curve distance (p < 0.05) throughout the entire MC volume compared to other popular deep learning networks. As a result, the Canal-Net achieved high consistent accuracy in 3D segmentations of the entire MC in spite of the areas of low visibility by the unclear and ambiguous cortical bone layer. Therefore, the Canal-Net demonstrated the automatic and robust 3D segmentation of the entire MC volume by improving structural continuity and boundary details of the MC in CBCT images.

## Introduction

The mandibular canal (MC) is an important mandibular structure that supplies sensation to the lower teeth, chin, and lower lip^1^. Any injury to the MC can lead to temporary or permanent damage resulting in sensory disturbance sequelae such as paresthesia, hypoesthesia, and dysesthesia, which affects speech, mastication, and quality of life^2–5^. Therefore, knowing the exact localization of the MC is essential in planning appropriate oral-maxillofacial surgeries such as implant placement and third molar extractions^6,7^. In preoperative assessments and surgical planning in dental clinics, panoramic radiographs are used as a standard dental imaging tool^8,9^, but presents limitations in that it is challenging to determine the actual 3D rendering of the entire canal structure as the panoramic radiograph only shows the canal in a single view^10^. Therefore, additional investigations using CT may be recommended to verify the exact position of the canal in a 3D view in accordance with the as low as reasonably achievable (ALARA) principle^8^. Due to the advantages of CBCT such as a lower radiation dose, inexpensive image acquisition cost, and high spatial resolution, CBCT has been widely used in dental clinics for 3D diagnosis and treatment planning in the field of oral and maxillofacial surgery^11–13^. However, the manual segmentation of the MC that is generally performed using 3D cross-sectional slices in CBCT images is time-consuming and labor-intensive^10,14^. In addition, the ambiguous cortical bone layer surrounding the canal and the unclear medulla pattern also makes it difficult to distinguish the entire MC because of the lower contrast of CBCT images^15^. Therefore, automatic segmentation of the MC is required to alleviate the workload of dental clinicians by overcoming the limitations of CBCT images.

Among studies for automatic MC segmentation in CBCT images, atlas-based segmentation (ARS) and statistical shape model (SSM) methods have been proposed as two conventional representatives of MC segmentation methods^16–18^. The SSM method utilized the prior knowledge of shape models to perform MC segmentation^17,18^. This prior knowledge was required to reconstruct a 3D model of CBCT images, which highly affects the segmentation result^17,18^. On the other hand, the ARS method only requires the atlas image for MC segmentation, which is independent of prior knowledge^16^. However, both the SSM and ARS methods exhibit a limitation in dealing with new forms of data beyond the predefined standard since they depend on prior knowledge or other preprocessing techniques^16–18^. Recently, deep learning methods have been widely used for the detection^19–21^, classification^22–24^, segmentation^25,26^, and enhancement^27,28^ of medical and dental images. Several convolutional neural networks (CNN) such as 3D U-Net, a type of deep learning method, were used for MC segmentation in CBCT images exhibiting a high accuracy of segmentation^10,14^. However, these CNNs failed to segment the MC with high consistent accuracy throughout its entire range because of the occasional unclear and ambiguous cortical bone layer caused by the overall lower contrast of CBCT images^10,14^. CNNs for the segmentation of the entire MC exhibited lower accuracy around the mandibular and mental foramens compared to other parts of the canal^10,14^ since discrimination of the canal from its surroundings became increasingly less clear towards the mental foramen region, and visibility of the MC clearly decreased on cross-sectional images of more distal regions of the MC^15^. Precise MC segmentation with high consistent accuracy throughout the entire MC is essential for avoiding nerve injury in oral and maxillofacial surgeries such as mandibular osteotomy and implant surgery^29^.

We hypothesized that a deep learning model yielded more robust 3D segmentation of the entire MC volume in CBCT images by learning the spatio-temporal features and structural continuity of the MC volume. In this study, we proposed a continuity-aware contextual network (Canal-Net) for the automatic and robust 3D segmentation of the MC with high consistent accuracy throughout the entire MC volume in CBCT images and compared our network with other networks in terms of volumetric accuracy over the entire canal. Our main contributions were as follows: (1) We proposed a continuity-aware contextual network (Canal-Net) that was robust to ambiguous or unclear cortical bone regions of the MC and lower contrast of CBCT images in 3D segmentations of the entire MC. (2) We applied bidirectional convolutional LSTM (ConvLSTM) in order to learn 3D anatomical contextual information of the MC by incorporating spatio-temporal features. (3) We used a multi-task learning framework with multi-planar projection losses (MPL) in three anatomical planes in order to evaluate the global structural continuity of the MC.

## Materials and methods

### Data acquisition and preparation

We included 50 patients (27 females and 23 males; mean age 25.56 ± 6.73 years) who underwent dental implant surgeries or third molar extractions at the Seoul National University Dental Hospital (2019–2020). The patients had different mandibular canal shapes with various dental conditions including the metallic crowns and implants. The patient data were obtained at 80 kVp and 8 mA using CBCT (CS9300; Carestream Health, New York, USA). The CBCT images had dimensions of 841 × 841 × 289 pixels, voxel sizes of 0.2 × 0.2 × 0.2 mm^3^, and 16-bit depth. This study was performed with approval from the institutional review board of the Seoul National University Dental Hospital (ERI18001). The ethics committee approved the waiver for the informed consent because this was a retrospective study. The study was performed in accordance with the Declaration of Helsinki.

The mandibular canals including the surrounding cortical bone was manually annotated by an oral and maxillofacial radiologist using a software (3D Slicer for Windows 10, Version 4.10.2; MIT, Massachusetts, USA)^30^. We used the cropped images consisting of 200 slices of 128 × 128 pixels that were centered at the left and right mandibular regions in order to reduce the memory requirement. Zero-padding was performed to maintain the input volume of the same length for all patients showing the mandibular canals of different lengths. For deep learning, we prepared 60 volumes from 30 patients for the training dataset, 20 from ten patients for the validation dataset, and 20 from ten patients for the test dataset where the right mandible images were horizontally flipped to match the left. We performed five-fold cross-validation, where each training cycle consisted of 60, 20, and 20 volumes for training, validation and test datasets, respectively.

We estimated the minimally required sample size to detect significant differences in the accuracy between the Canal-Net and the other networks, when both assessed the same subjects (CBCT images). We designed to capture a mean accuracy-difference of 0.05, and a standard deviation of 0.10 between the Canal-Net and the other networks. Based on an effect size of 0.5, a significance level of 0.05, and a statistical power of 0.80, we obtained a sample size of N = 128 (G* Power for Windows 10, Version 3.1.9.7; Universität Düsseldorf, Germany). Eventually, we split the CBCT dataset of 2D images into 10,185, 2546, and 3183 for training, validation, and test datasets, respectively.

### Continuity-aware contextual network (Canal-Net)

**Figure 1:**
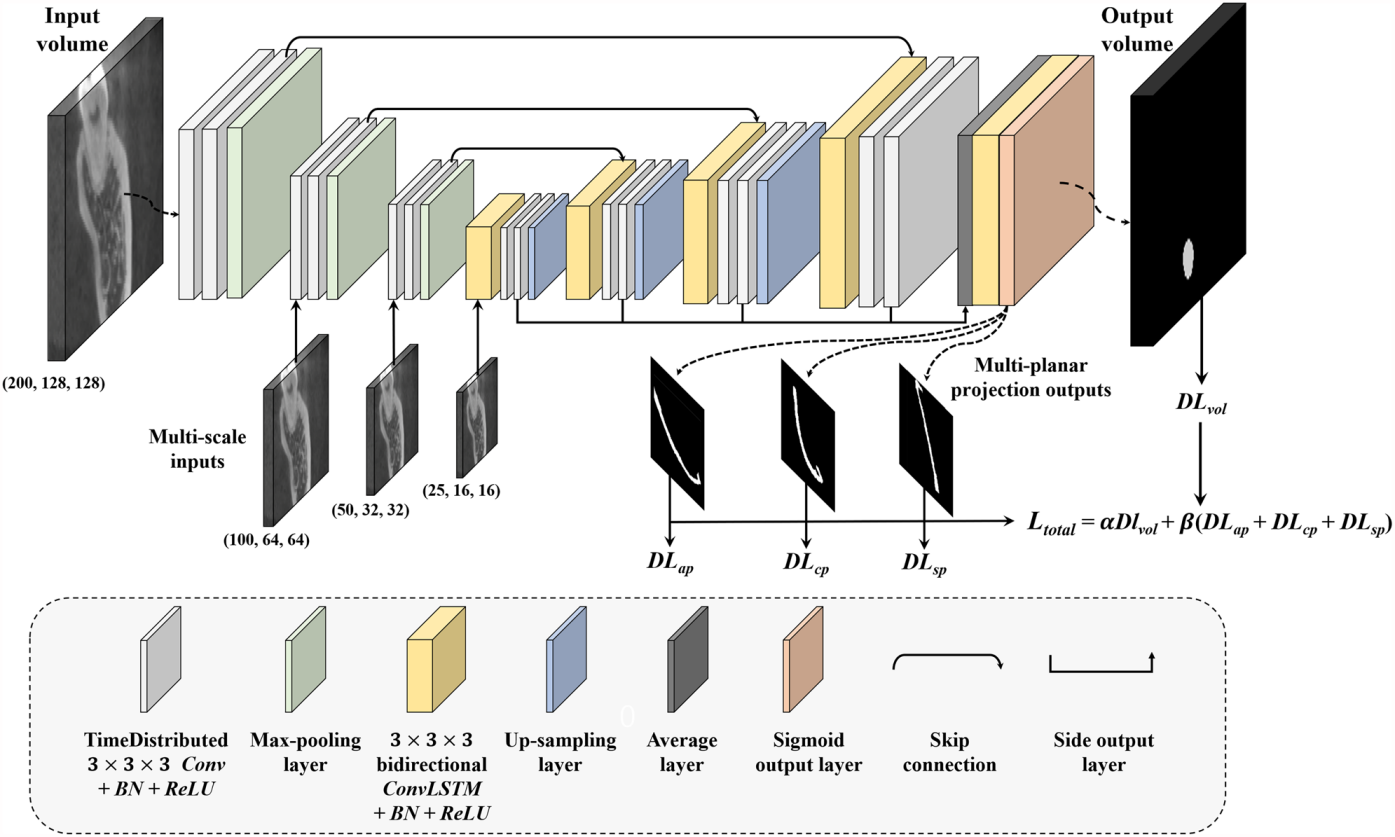
The Canal-Net architecture with a 3D encoder-decoder under a multi-task learning framework consisting of time-distributed convolution blocks, multi-scale inputs, skip connection, and bidirectional convolutional LSTM (ConvLSTM) with side-output layers. The bidirectional ConvLSTM was utilized to capture anatomical context information, and a multi-task learning approach was performed to learn overall MC volume and structural continuity.

We designed a continuity-aware contextual network (Canal-Net) which had 3D encoder-decoder architecture under a multi-task learning framework consisting of time-distributed convolution blocks, multi-scale inputs^31^, skip connections, and bidirectional convolutional LSTM (ConvLSTM) with side-output layers^31,32^ (Fig. 1). The bidirectional ConvLSTM was used to capture anatomical context information in concatenated feature maps extracted from the corresponding encoding path and the previous decoding up-sampling layer. A multi-task learning approach was adopted to simultaneously output the entire MC volume and its 2D multi-planar projections in three anatomical planes, which helped the network learn the overall MC volume and structural continuity (multi-planar projection outputs and the output volume in Fig. 1). The network under multi-task learning was optimized in an end-to-end manner, where the MC segmentation output was generated directly from the input volumes of the CBCT images.

At the encoder, the time-distributed convolution blocks processed sequential information from 3D volumetric inputs as series of features for 2D slices^33^ (white blocks in Fig. 1). It was a typical convolution passed to a time-distributed wrapper that could be applied to every temporal frame of the input independently^33^. Convolutional blocks were comprised of two repeated modules of two 3 × 3 × 3 convolutions, batch normalization, ReLU, and 2 × 2 × 2 max-pooling at the encoder path. The number of feature maps gradually decreased from 128 to 64, 32, and 16. To mitigate spatio-temporal information loss caused by max-pooling operations, the multi-scale inputs down-sampled from the original input volume by 2 × 2 × 2 average pooling were concatenated at each level of the encoder (multi-scale inputs in Fig. 1).

At the decoder, the features from time-distributed convolutions at the encoder^33^ were concatenated with the corresponding up-sampling layer and fed to bidirectional ConvLSTM blocks (Skip connection and yellow blocks in Fig. 1). Long short-term memory (LSTM), one of the recurrent neural networks (RNN)^34^, was an efficient network for handling spatio-temporal data and was widely used in contextual processing such as natural language processing^35^ and video segmentation^36^. The internal matrix multiplication of the original LSTM was replaced by the convolution operation to maintain the input dimension in ConvLSTM^37^. The ConvLSTM blocks were composed of two repeated modules of two 3 × 3 × 3 bidirectional ConvLSTMs, batch normalization, ReLU, and 2 × 2 × 2 up-sampling at the decoder path. The number of feature maps gradually increased from 16 to 32, 64, and 128. The ConvLSTM captured 3D local anatomical contextual information more effectively by learning the spatio-temporal features of the 3D volumetric data^37^.

At the output layer, the averaged side-outputs generated from a local output map from every level of the decoder were merged and fed to the bidirectional ConvLSTM, which mitigated the gradient vanishing problem for encouraging the back-propagation of gradient flow (Side output and average layers in Fig. 1). The 3D volume loss and multi-planar projection losses (MPL) from the 2D projections simultaneously encouraged the network to learn the global structural continuity information of the canal under the multi-task learning framework. The MPL were calculated from the 2D projection maps of the output in three anatomical planes. The Dice similarity coefficient score (DSC) was used for the two loss functions^38^. The loss function ($L= \alpha {DL}_{vol}+\beta {({DL}_{ap}+ DL}_{cp}+{DL}_{sp})$ of the Canal-Net consisted of 3D volume loss (${DL}_{vol}$) for the entire canal volume, and the MPL as sum of the 2D projection losses in axial- (${DL}_{ap}$), coronal- (${DL}_{cp}$), and sagittal- (${DL}_{sp}$) planes, where α and β were constant weights for the 3D volume loss and the summation of the 2D projection map losses, respectively (equation of ${L}_{total}$ in Fig. 1). The weights of α and β were optimized for the best performance through an ablation study. The weights of 0.7 and 0.3 for the 3D volume loss and MPL, respectively, exhibited the best performance compared to other weight options (Table 1).

**Table 1:** Comparison of Dice similarity coefficient scores (DSC) for α, weight of 3D volume loss, and β, weight of the MPL in axial, coronal, and sagittal planes to determine the optimal loss weights for α and β for the Canal-Net.

Loss weight	3D volume	2D axial	2D coronal	2D sagittal
α = 0.1, β = 0.9	0.84 ± 0.14	0.92 ± 0.07	0.91 ± 0.17	0.91 ± 0.14
α = 0.2, β = 0.8	0.85 ± 0.12	0.91 ± 0.10	0.90 ± 0.12	0.89 ± 0.13
α = 0.3, β = 0.7	0.84 ± 0.16	0.90 ± 0.14	0.87 ± 0.17	0.88 ± 0.17
α = 0.4, β = 0.6	0.83 ± 0.16	0.90 ± 0.15	0.83 ± 0.19	0.88 ± 0.16
α = 0.5, β = 0.5	0.86 ± 0.13	0.91 ± 0.10	0.88 ± 0.16	0.91 ± 0.11
α = 0.6, β = 0.4	0.84 ± 0.14	0.89 ± 0.13	0.86 ± 0.17	0.89 ± 0.13
α = 0.7, β = 0.3	0.87 ± 0.05	0.93 ± 0.07	0.91 ± 0.14	0.94 ± 0.08
α = 0.8, β = 0.2	0.87 ± 0.09	0.92 ± 0.09	0.91 ± 0.14	0.92 ± 0.09
α = 0.9, β = 0.1	0.86 ± 0.13	0.91 ± 0.10	0.87 ± 0.16	0.92 ± 0.10

The proposed networks were trained using an Adam optimizer, and the learning rate of 0.00025 was reduced on plateau by a factor of 0.5 every 25 epochs in 300 epochs with the batch size of 1. They were implemented with Python3 based on Keras with a Tensorflow backend using a single NVIDIA Titan RTX GPU 24G.

### Performance evaluation of Canal-Net for MC segmentation

We compared the performance of the MC segmentation by Canal-Net with those by other networks of 2D U-Net^39^, SegNet^40^, 3D U-Net^41^, 3D U-Net with MPL (MPL 3D U-Net), and 3D U-Net with ConvLSTM (ConvLSTM 3D U-Net). To evaluate the performances quantitatively, we compared the 2D segmentation performance metrics of the Dice similarity coefficient score ($DSC=\frac{2TP}{2TP+FN+FP}$), Jaccard index ($JI=\frac{TP}{TP+FN+FP}$), precision ($PR=\frac{TP}{TP+FP}$), recall ($RC=\frac{TP}{TP+FN}$) among networks, where TP, FP, and FN denoted true positives, false positives, and false negatives, and also 3D volumetric performance metrics of volume of error ($VOE=1- \frac{{V}_{gt}\cap {V}_{pred}}{{V}_{gt}\cup {V}_{pred}}$) and relative volume difference ($RVD=\frac{|{V}_{gt}-{V}_{pred}|}{{V}_{gt}}$), where ${V}_{gt}$ and ${V}_{pred}$ represented the number of voxels for the ground truth and for the predicted volume, respectively. We also evaluated the mean curve distance ($MCD=\frac{\sum_{t\epsilon C\left({V}_{gt}\right)}dist\left(t,C\left({V}_{pred}\right)\right)}{|C\left({V}_{gt}\right)|}$)), where $dist\left(x, Y\right)={min}_{y\epsilon Y}\{{|x-y|}^{2}\}$, and $\mathrm{t}$ denotes coordinates of a ground truth voxel^14^, and $C(\cdot )$ is an operation which extracted the center curve line through skeletonization for the set of voxels^14^. The higher values of DSC, JI, PR, and RC, and the lower values of VOE, RVD, and MCD indicated better segmentation performance. We used paired two-tailed t-tests to compare performances between Canal-Net and others (SPSS Statistics for Windows 10, Version 26.0; IBM, Armonk, New York, USA). The statistical significance level was set at 0.05. We also performed the Bland–Altman analysis to analyze the bias and agreement limits of the used segmentation models between the number of pixels of ground truth and prediction results.

## Results

The performances of Canal-Net, convLSTM 3D U-Net, MPL 3D U-Net, 3D U-Net, SegNet, and 2D U-Net were evaluated for a total of 20 mandibular canals not used for training. Among them, convLSTM 3D U-Net, MPL 3D U-Net, and 3D U-Net were evaluated to demonstrate the effectiveness of the corresponding components in Canal-Net, while the other networks were used for performance comparisons between 2 and 3D CNN-based approaches. In addition, the Canal-Net was evaluated for the impacts of the weights of *α* and *β* on 3D volume loss and MPL, respectively. The Canal-Net with loss weights of *α* = 0.7 and *β* = 0.3 achieved the best segmentation performance of 0.87, 0.93, 0.91, and 0.94 DSC for 3D volume, axial, coronal, and sagittal planes, respectively (Table 1).

**Figure 2:**
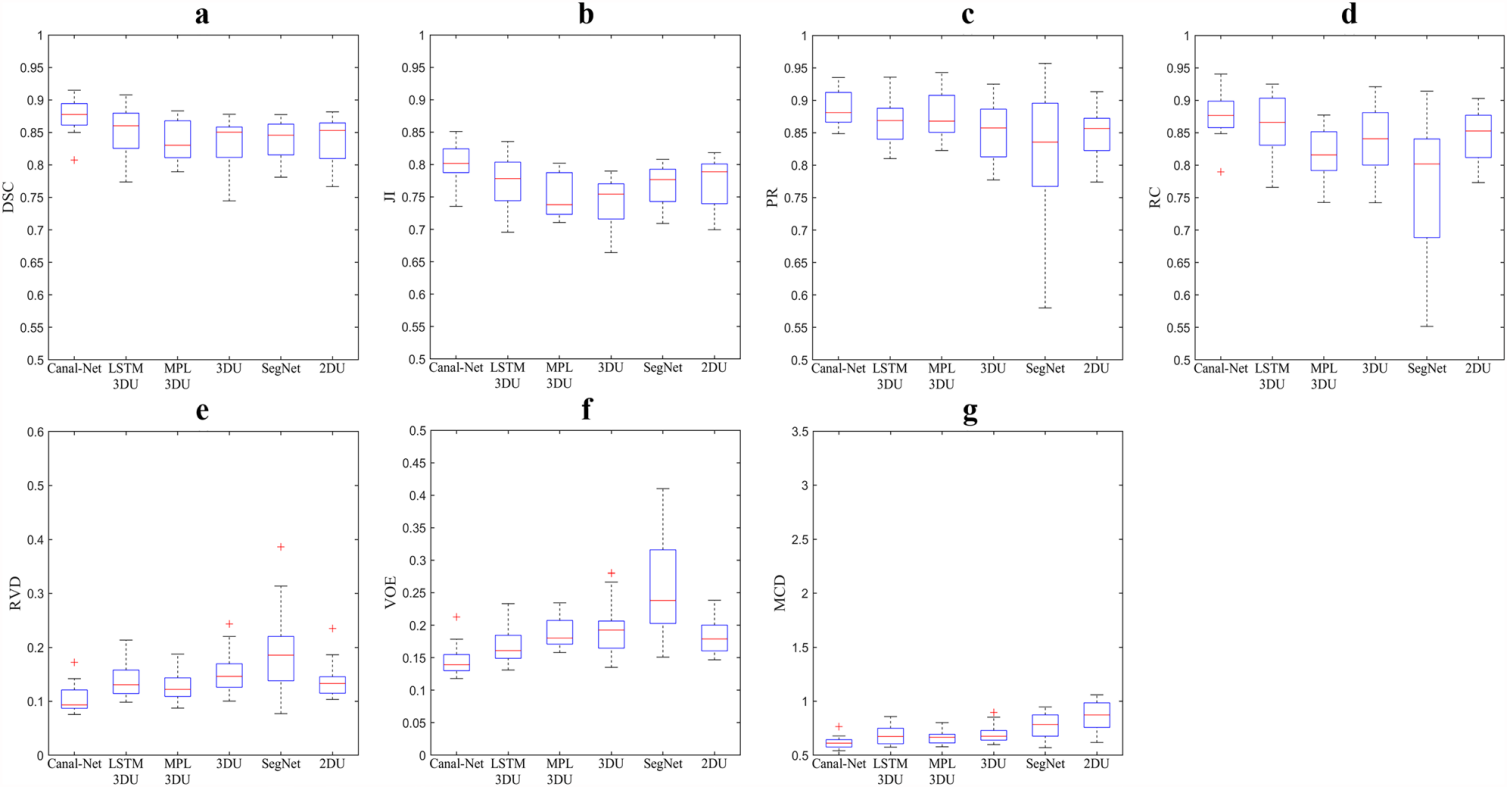
The boxplots of segmentation performance results of the (**a**) Dice similarity coefficient score (DSC), (**b**) Jaccard index (JI), (**c**) precision (PR), (**d**) recall (RC), (**e**) relative volume difference (RVD), (**f**) volume of error (VOE), and (**g**) mean curve distance (MCD) for the deep learning networks, Canal-Net, ConvLTSM 3D U-Net (LSTM 3DU), MPL 3D U-Net (MPL 3DU), 3D U-Net (3DU), SegNet, and 2D U-Net (2DU). Each box contains the first and third quartile of data. The medians are located inside of the boxes, visualized as red lines. The whiskers are extended above and below each box in ± 1.5 times the interquartile range (IQR), and the outliers are visualized as red + marks defining values 1.5 IQR away from the box.

Table 2 shows the quantitative results of the segmentation performance by the networks. The performances of Canal-Net, ConvLSTM 3D U-Net, MPL 3D U-Net, 3D U-Net, SegNet, and 2D U-Net were compared using 20 total mandibular canals. The Canal-Net achieved the highest values of 0.87 DSC (p < 0.05), 0.80 JI (p < 0.05), 0.89 PR (p = 0.05), and 0.88 RC (p = 0.05) in 2D performance metrics, and also the lowest values of 0.14 RVD (p < 0.05), 0.10 VOE (p < 0.05), and 0.62 MCD (p < 0.05) in 3D performance metrics (Table 2). The Canal-Net outperformed all the other networks in DSC, JI, PR, RC, RVD, and VOE, and significantly so in MCD (p < 0.05) (Table 2). The performance of the networks is also plotted in boxplots (Fig. 2). The Canal-Net achieves the higher performances than the other networks with a smaller dispersion of data, shorter length of whiskers, and rare existence of outliers (Fig. 2).

**Table 2:** Mean (SD) dice similarity coefficient score (DSC), Jaccard index (JI), precision (PR), recall (RC), volume of error (VOE), relative volume difference (RVD), and mean curve distance (MCD) by Canal-Net (ours), ConvLSTM 3D U-Net (ours), MPL 3D U-Net (ours), 3D U-Net, SegNet, and 2D U-Net Net by five-fold cross-validation.

	DSC	JI	PR	RC	RVD	VOE	MCD (mm)
Canal-Net	0.87 ± 0.05	0.80 ± 0.06	0.89 ± 0.06	0.88 ± 0.06	0.14 ± 0.04	0.10 ± 0.04	0.62 ± 0.10
ConvLSTM 3D U-Net	0.85 ± 0.08*	0.77 ± 0.08*	0.87 ± 0.08*	0.86 ± 0.09*	0.17 ± 0.05*	0.13 ± 0.05*	0.66 ± 0.12*
MPL 3D U-Net	0.84 ± 0.06^†^	0.75 ± 0.07^†^	0.88 ± 0.07^†^	0.82 ± 0.08^†^	0.19 ± 0.04^†^	0.14 ± 0.06^†^	0.69 ± 0.15^†^
3D U-Net	0.83 ± 0.07^‡^	0.74 ± 0.07^‡^	0.85 ± 0.08^‡^	0.84 ± 0.09^‡^	0.19 ± 0.05^‡^	0.15 ± 0.07^‡^	0.69 ± 0.13^‡^
SegNet	0.84 ± 0.06^+^	0.77 ± 0.06^+^	0.85 ± 0.06^+^	0.85 ± 0.07^+^	0.18 ± 0.04^+^	0.14 ± 0.05^+^	0.78 ± 0.19^+^
2D U-Net	0.84 ± 0.07^Φ^	0.77 ± 0.07^Φ^	0.85 ± 0.07^Φ^	0.84 ± 0.08^Φ^	0.18 ± 0.04^Φ^	0.14 ± 0.05^Φ^	0.87 ± 0.22^Φ^

*Significant difference between Canal-Net and ConvLSTM 3D U-Net (p < 0.05).

^†^Between Canal-Net and MPL 3D U-Net (p < 0.05).

^‡^Between Canal-Net and 3D U-Net (p < 0.05).

^+^Between Canal-Net and SegNet (p < 0.05).

^Φ^Between Canal-Net and 2D U-Net (p < 0.05).

**Figure 3:**
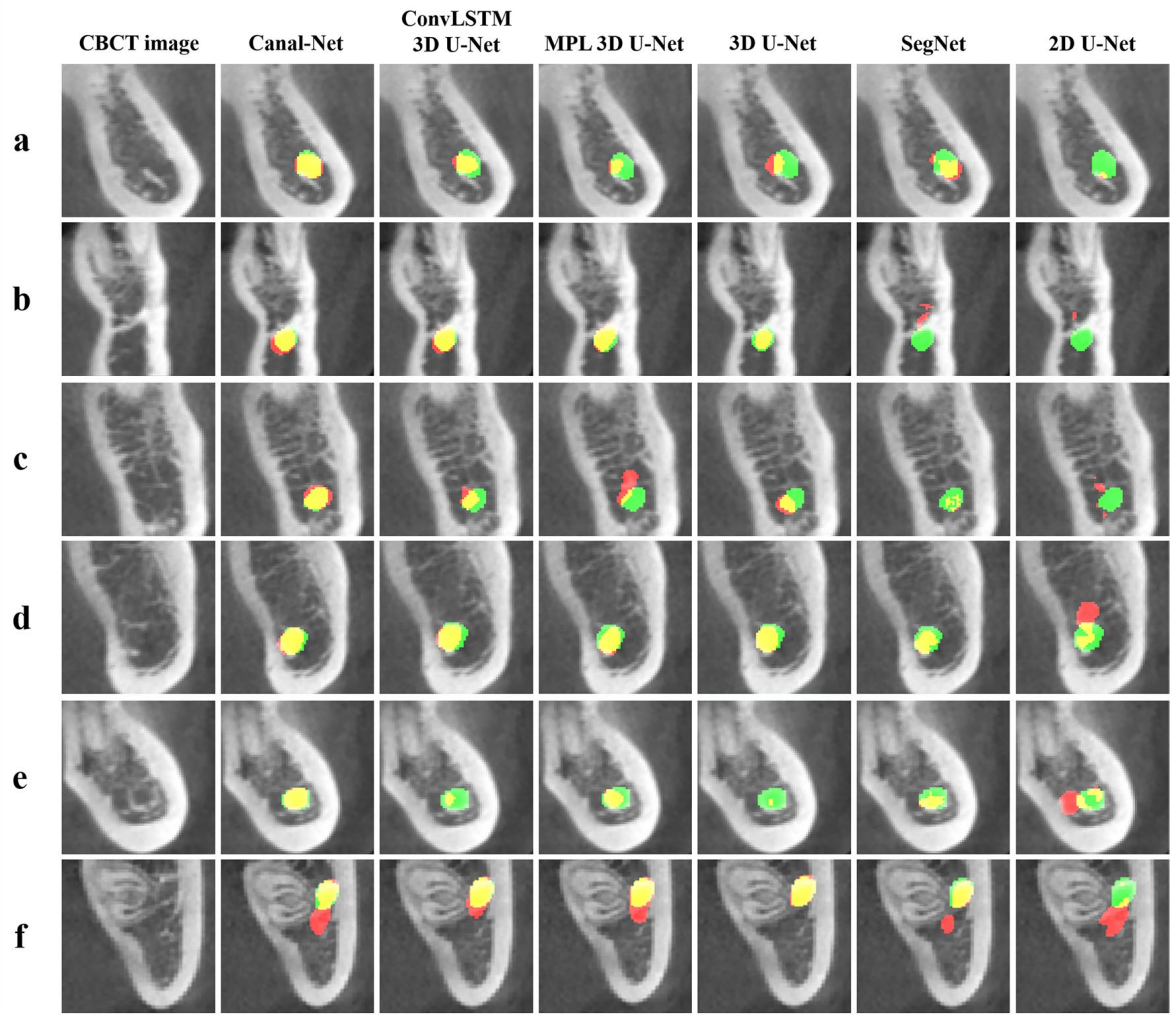
The segmentation results by Canal-Net (ours), ConvLSTM 3D U-Net (ours), MPL 3D U-Net (ours), 3D U-Net, SegNet, and 2D U-Net. The yellow, green, and red areas indicate the true positive, false negative, and false positive, respectively. The segmentation results for the MC with (**a–c**) low visibility and (**d,e**) containing the metallic crown and implant fixture, and for (**f**) the outlier of the Canal-Net in the boxplot in Fig. 2.

**Figure 4:**
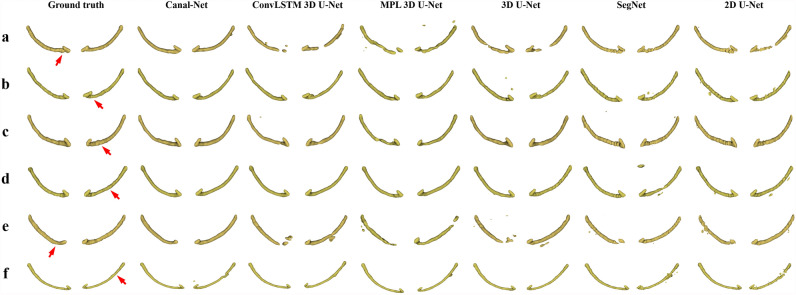
The 3D reconstructed whole canal from the ground truth and segmentation results by Canal-Net (ours), ConvLSTM 3D U-Net (ours), MPL 3D U-Net (ours), 3D U-Net, SegNet, and 2D U-Net from the top to bottom (**a–f**) from the same canals shown in Fig. 3a–f. The red arrows indicate the position of the corresponding image slice in Fig. 3.

In Fig. 3, the Canal-Net exhibited more accurate predictions with more true positives (yellow) and less false positives (red) and false negatives (green) compared to the other networks for MCs with unclear and ambiguous cortical bone layers and metallic objects in CBCT images of lower contrast (Fig. 3a–e). In the 3D segmentation results, the Canal-Net also demonstrated better prediction results with less false positives and false negatives compared to the other networks in the mental foramen area of the various MC shapes (Fig. 4a–e). Furthermore, only a few cases as outliers for the results of the Canal-Net were observed due to other causes such as the presence of a third molar beside the MC (Figs. 3f, 4f). The Canal-Net predicted more accurately the entire MC volume, and demonstrated improved structural continuity and boundary details of the MC from the mental foramen to the mandibular foramen compared to the other networks (Fig. 4a–e).

**Figure 5:**
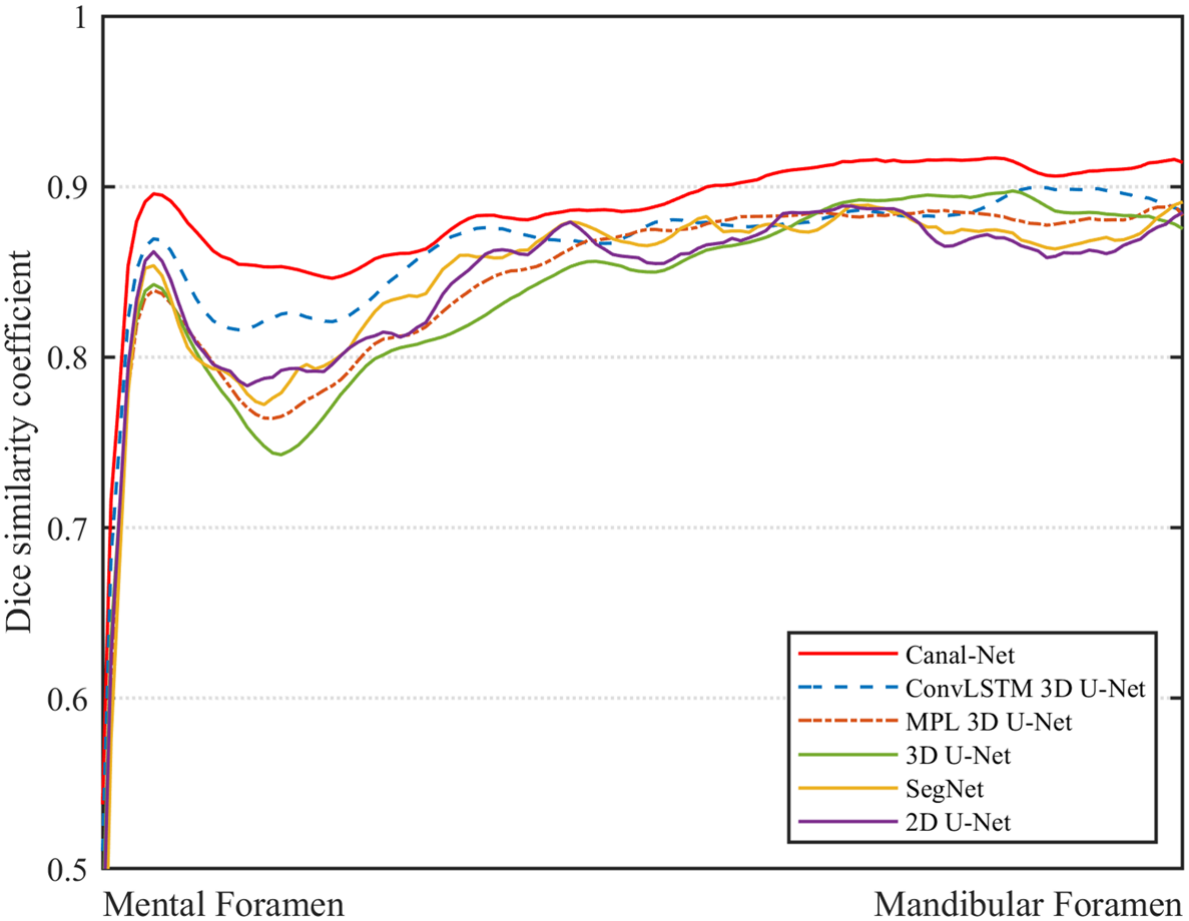
The line plots of dice similarity coefficient score (DSC) from the mental foramen to the mandibular foramen for Canal-Net (ours), ConvLSTM 3D U-Net (ours), MPL 3D U-Net (ours), 3D U-Net, SegNet, and 2D U-Net.

**Figure 6:**
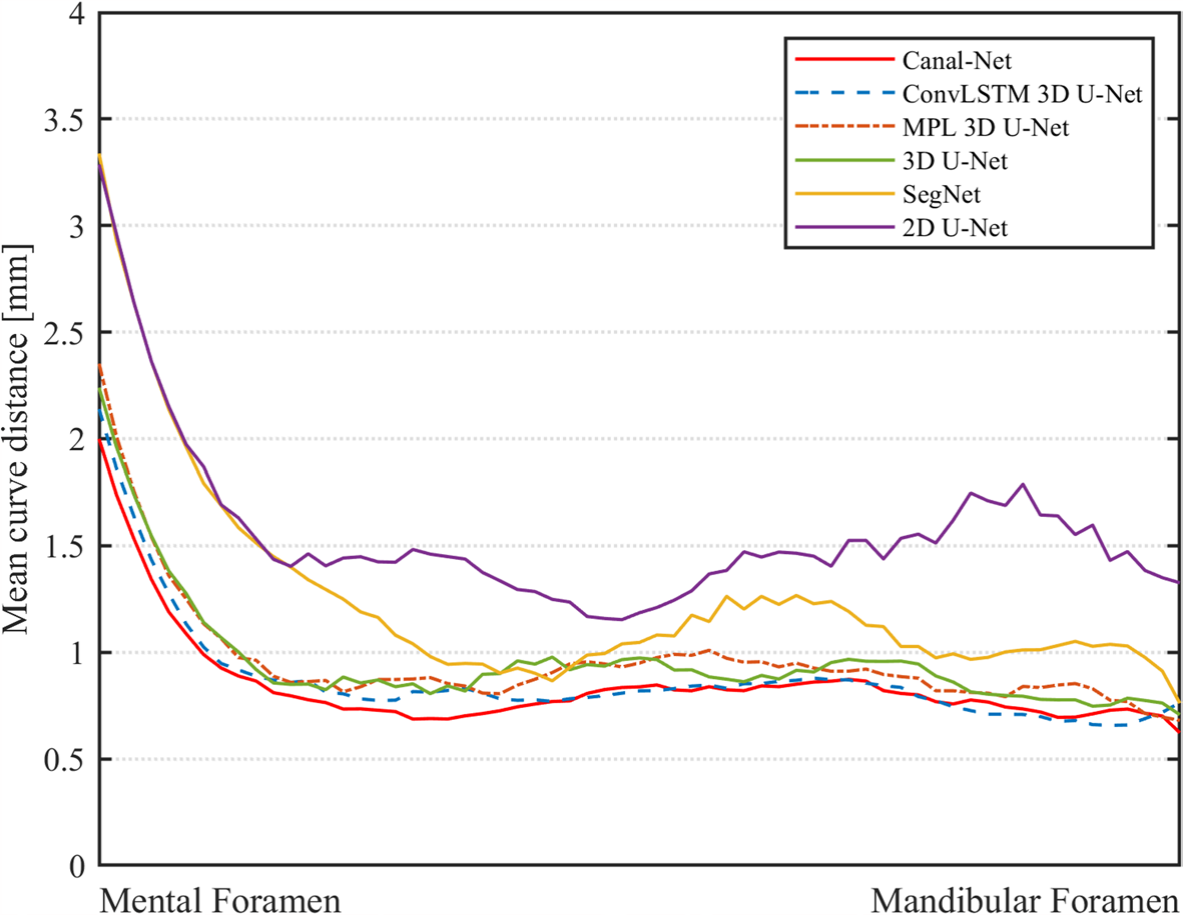
The line plots of mean curve distance (MCD) from the mental foramen to the mandibular foramen for Canal-Net (ours), ConvLSTM 3D U-Net (ours), MPL 3D U-Net (ours), 3D U-Net, SegNet, and 2D U-Net.

**Figure 7:**
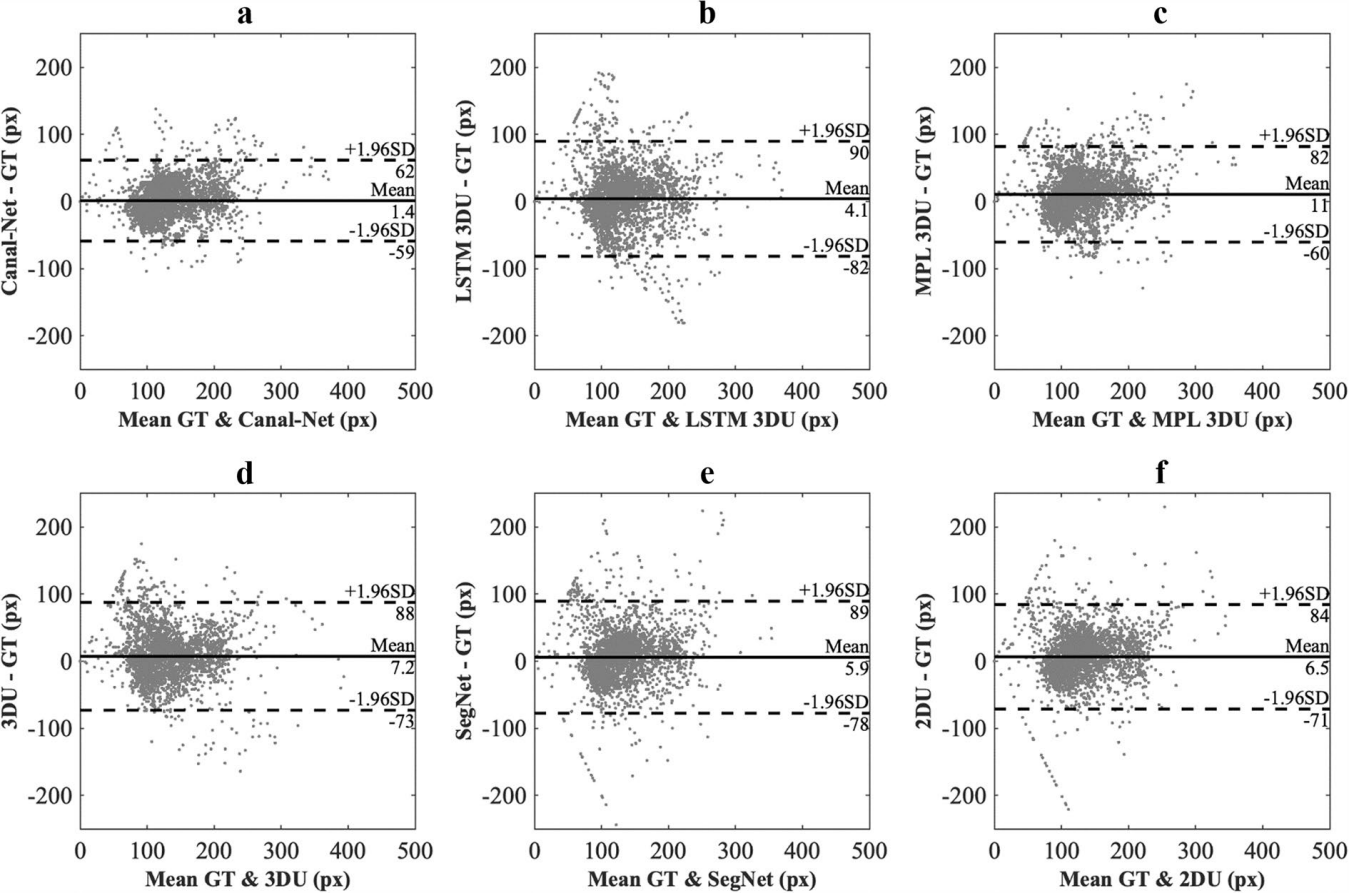
The Bland–Altman plots between the ground (GT) and prediction results from the (**a**) Canal-Net, (**b**) convLSTM 3D U-Net (LSTM 3DU), (**c**) MPL 3D U-Net (MPL 3DU), (**d**) 3D U-Net (3DU), (**e**) SegNet, and (**f**) 2D U-Net (2DU).

The DSC and MCD for the whole test dataset were plotted from the mental foramen to the mandibular foramen, and the 3D networks generally exhibited less variations of the performances compared to the 2D networks (Figs. 5 and 6). The Canal-Net demonstrated the most consistent performances with the smallest fluctuations of true segmentation compared to the other networks throughout the entire MC volume (Figs. 5 and 6). As a result, the Canal-Net represented the best 3D segmentation accuracies of RVD, VOE, and MCD throughout the entire MC volume among the networks (Table 2). The Bland–Altman plot between the ground truth and prediction results from the Canal-Net showed higher linear relationships and better agreement limits than those from the other networks (Fig. 7). Therefore, the Canal-Net represented more accurate and robust MC segmentation performance of the entire MC compared to the other networks.

## Discussion

In this study, we proposed a continuity-aware contextual network (Canal-Net) which learned 3D local anatomical contextual information and the global continuity of the MC complementally in order to segment the MC with high consistent accuracy throughout the entire MC volume in cone-beam CT (CBCT) images. We employed time-distributed convolution layers for handling time-distributed sequential features with multi-scale inputs at the encoder path^33^, and bidirectional ConvLSTM layers for extracting spatio-temporal features at the decoder path^37^. The Canal-Net was able to learn the local anatomical variations of the MC by incorporating the spatio-temporal features effectively, and the global structural continuity information of the MC under the multi-task learning framework, complementally. The Canal-Net used optimized weights for 3D volume loss and multi-planar projection losses in multi-task learning. Therefore, the Canal-Net improved the performance of automatic segmentation of the MC by combining anatomical context information and global structural continuity information, resulting in higher consistent accuracy throughout the entire MC volume in CBCT images.

We compared the Canal-Net with other popular segmentation networks such as 2D U-Net, SegNet, and 3D U-Net, and also with our MPL 3D U-Net and ConvLSTM 3D U-Net for MC segmentation. In performances of MC segmentation in CBCT images, 2D U-Net and SegNet exhibited lower accuracies compared to the 3D networks, generally. False negatives and positives were observed at a higher rate around the mental foramen area with ambiguous or unclear cortical bone layers. Since the 2D networks were not able to learn the 3D contextual features of the MC volume in CBCT images, the 2D networks exhibited coarser 3D segmentation volumes with more fluctuations of 3D performance accuracy from the mental to the mandibular foramen regions. In terms of learning 3D spatial contextual information between image slices of the 3D anatomical structures, 3D U-Net was generally expected to generate more accurate segmentation results compared to 2D networks^41^. In the present study, the 3D U-Net predicted the more accurate segmentation of the MC with fewer false positives and negatives compared to the 2D U-Net and SegNet. However, the 3D U-Net had still limitations in segmenting the MC regions with unclear cortical bone layers accurately by only learning 3D spatial information between image slices, and exhibited inaccurate segmentation results with disconnections around the mental foramen area.

Both MPL 3D U-Net and ConvLSTM 3D U-Net demonstrated better segmentation results than 3D U-Net in different aspects. The MPL 3D U-Net showed an improved travel course of the MC compared to 3D U-Net because its spatial information was complemented with the global structural continuity information by learning through multi-planar projections. Although the structural continuity of the MC volume was improved by multi-task learning, the MPL 3D U-Net exhibited difficulties in producing segmentation boundaries in detail around the mental foramen area. On the other hand, the ConvLSTM learned anatomical context information through spatio-temporal features, and the MC volume showed smooth boundaries with more consistent accuracies even in unclear cortical bone layer regions in the CBCT images. Therefore, the Canal-Net demonstrated the most accurate segmentation of the entire MC volume compared to the other networks by simultaneously learning global structural continuity through MPL, and anatomical context information through ConvLSTM. Compared with previous studies using 3D U-Net^10,14^, our Canal-Net achieved 0.87 of DSC and 0.80 of the mean intersection of union (IoU) while two previous studies reported 0.58 of DSC^10,14^ and 0.58 of mean IoU^10,14^. Compared with the previous studies^10,14^, the Canal-Net showed substantially enhanced performance of the MC segmentation in CBCT images.

In the Canal-Net, the MPL provided global structural continuity from three anatomical projection maps with ConvLSTM anatomical context information by spatio-temporal features, complementally. In the MC areas of low visibility with ambiguous or unclear cortical bone layers in CBCT images, the Canal-Net exhibited the best outcomes with continuous and consistent MC volumes from the mental to mandibular foramens. The Canal-Net especially surpassed other networks by showing continuous MC volumes around the mental foramen area where the visibility of the MC tended to diminish^15^, and in areas affected by metallic objects such as implant fixtures or dental crowns in CBCT images. As a result, the Canal-Net demonstrated the most robust MC segmentation with high consistent DSC throughout the entire MC volume in CBCT images.

The primary reason for improved segmentation performance by Canal-Net was that its network architecture was constructed to complementally learn the 3D anatomical context information of the MC by the spatio-temporal features from the bidirectional ConvLSTM layers and the global structural continuity information by MPL. In the Canal-Net, the complementary context information was successfully learned in the proposed framework, leading to maintaining continuous and consistent MC volumes from the mental to the mandibular foramen areas. The proposed learning process has several advantages. First, it could increase the discriminative capability of intermediate feature representations with multiple regularizations on disentangling subtly correlated tasks^48^, potentially improving the robustness of the segmentation performance. Second, in the application of MC segmentation, the multi-task learning framework could also provide complementary context information that would serve well to segment the MC maintaining overall continuous and consistent volumes. This could improve the performance accuracy of MC segmentations substantially, especially in MC regions with ambiguous or unclear cortical bone layers in lower contrast CBCT images.

The accurate identification of the whole MC structure in the mandible is an essential prerequisite for the preoperative planning of third molar extractions and implant surgeries to avoid any surgical complications^7^. However, the exact recognition of the entire canal structure is considered to be a challenging and delicate task for several reasons^15^. CBCT, the most commonly used 3D dental imaging tool, has lower contrast than CT, which negatively affects the ability to distinguish MCs^10,42^. As a result, the low visibility of MCs, such as in ambiguous or unclear cortical bone regions, affects the structural continuity of MC segmentation in CBCT images^10,14^. Furthermore, the visibility of the MC itself is low due to variable cortications and bone densities of the canal wall, the diverse travel courses of the canal, and the spread of vessels and nerve branches^15,43–47^. The Canal-Net could be used in automatic and robust 3D segmentation of the MC structure for the preoperative planning of third molar extractions and implant surgeries to avoid any surgical complications when using CBCT images. The automatic segmentation of the MC volume by the Canal-Net could provide clinicians with accurate identification of the MC structure in the mandible with high consistent accuracy throughout the entire MC volume ranging from the mental foramen to the mandibular foramen while reducing time and effort.

However, our study had several limitations. First, as there was the problem of reducing the memory requirements for dealing with large amounts of data when using deep 3D networks running on the GPU, it was necessary to optimize the way the memory was used in order to maximize GPU utilization. Therefore, we used the cropped images with smaller dimensions than the original, and preprocessing of the images required additional time and labor. Second, our study had a potential limitation of generalization ability due to using internal data from a single organization. Overfitting of training a deep learning model, which resulted in the model learning statistical regularity specific to the training dataset, could negatively impact the model’s ability to generalize to a new dataset^49^. Although the proposed network did not show the presence of overfitting for the internal dataset in the five-fold cross-validation, it needs to be trained and evaluated using large datasets from multiple organizations or devices for generalization. Third, the results presented in this study were based on datasets from 50 patients. The proposed method needs to be evaluated for datasets from more patients with various dental restorations and implants. In future studies, we will improve the generalization ability and clinical efficacy of the Canal-Net by using large CBCT datasets acquired under various imaging conditions from multiple organizations or devices.

## Conclusions

In this study, we proposed a continuity-aware contextual network (Canal-Net) that was robust to ambiguous or unclear cortical bone regions of the MC and lower contrast of CBCT images in 3D segmentations of the entire MC. The Canal-Net was designed based on a 3D U-Net with the ConvLSTM under the multi-task learning framework using MPL in order to complementally learn anatomical contexts and global structural continuity information. As a result, the Canal-Net achieved substantially enhanced performances compared to other networks such as 2D U-Net, SegNet, 3D U-Net, MPL 3D U-Net, and ConvLSTM 3D U-Net in 2D and 3D performances. Furthermore, Canal-Net demonstrated automatic and robust 3D segmentation of the entire MC volume by improving structural continuity and boundary details of the MC in CBCT images. The Canal-Net could be contributed to accurate and automatic identification of the MC structure for the preoperative planning of third molar extractions and implant surgeries to avoid any surgical complications.

## Data Availability

The datasets generated and/or analyzed during the current study are not publicly available due to the restriction by the Institutional Review Board (IRB) of Seoul National University Dental Hospital in order to protect patients’ privacy but are available from the corresponding author on reasonable request. Please contact the corresponding author for any commercial implementation of our research.
