# Author Correction: Canal-Net for automatic and robust 3D segmentation of mandibular canals in CBCT images using a continuity-aware contextual network

**DOI:** 10.1038/s41598-022-25677-2

**Published:** 2022-12-07

**Authors:** Bo-Soung Jeoun, Su Yang, Sang-Jeong Lee, Tae-Il Kim, Jun-Min Kim, Jo-Eun Kim, Kyung-Hoe Huh, Sam-Sun Lee, Min-Suk Heo, Won-Jin Yi

**Affiliations:** 1 Interdisciplinary Program in Bioengineering, Graduate School of Engineering, Seoul National University, Seoul, Korea; 2 Department of Applied Bioengineering, Graduate School of Convergence Science and Technology, Seoul National University, Seoul, Korea; 3 Vision AI Business Team, LG CNS, Seoul, Korea; 4 Department of Periodontology, School of Dentistry and Dental Research Institute, Seoul National University, Seoul, Korea; 5 Department of Electronics and Information Engineering, Hansung University, Seoul, Korea; 6 Department of Oral and Maxillofacial Radiology and Dental Research Institute, School of Dentistry, Seoul National University, Seoul, Korea

Correction to: *Scientific Reports* 10.1038/s41598-022-17341-6, published online 05 August 2022

The Acknowledgment section in the original version of this Article was incorrect.

“This work was supported by the Korea Medical Device Development Fund Grant funded by the Korean Government (The Ministry of Science and ICT, The Ministry of Trade, Industry, and Energy, The Ministry of Health & Welfare, and The Ministry of Food and Drug Safety) (1711137883, KMDF_PR_20200901_0011, 1711138289, RS-2020-KD00014). This work was also supported by a Seoul National University Research Grant in 2021.”

now reads:

“This work was supported by a Seoul National University Research Grant in 2021. This work was also supported by the Korea Medical Device Development Fund Grant funded by the Korean Government (The Ministry of Science and ICT, The Ministry of Trade, Industry, and Energy, The Ministry of Health & Welfare, and The Ministry of Food and Drug Safety) (Project Number: 1711174552, KMDF_PR_20200901_0147 and Project Number: 1711174543, RS-2020-KD000011).”

The original Article has been corrected.

